# Gender Differences in the Distribution of Creativity Scores: Domain-Specific Patterns in Divergent Thinking and Creative Problem Solving

**DOI:** 10.3389/fpsyg.2021.626911

**Published:** 2021-03-04

**Authors:** Wu-jing He, Wan-chi Wong

**Affiliations:** ^1^Department of Special Education and Counselling, The Education University of Hong Kong, Tai Po, Hong Kong; ^2^Integrated Centre for Wellbeing, The Education University of Hong Kong, Tai Po, Hong Kong; ^3^Department of Educational Psychology, The Chinese University of Hong Kong, Hong Kong, Hong Kong

**Keywords:** gender differences, greater male variability, creativity, divergent thinking, creative problem solving, variability analyses, measurement invariance

## Abstract

The present study examined gender differences in the distribution of creative abilities through the lens of *the greater male variability hypothesis*, which postulated that men showed greater interindividual variability than women in both physical and psychological attributes (Ellis, 1894/1934). Two hundred and six (51.9% female) undergraduate students in Hong Kong completed two creativity measures that evaluated different aspects of creativity, including: (a) a divergent thinking test that aimed to assess idea generation and (b) a creative problem-solving test that aimed to assess restructuring ability. The present findings extended the research of greater male variability in creativity by showing that men generally exhibited greater variance than women in the overall distribution of the creativity scores in both divergent thinking and creative problem solving, despite trivial gender differences in mean scores. The findings further enriched the discourse of the greater male variability hypothesis by showing interesting domain-specific gendered patterns: (1) greater male variability was more likely to occur in figural forms of creativity, with larger effect sizes, when compared to the variability in verbal forms of creativity; and (2) mixed gendered patterns were found in the upper tails of the creativity score distribution with respect to the verbal domain but not the figural one, despite greater male representation being consistently observed in the lower tail of the distribution. Possible underlying mechanisms and implications were discussed.

## Introduction

Gender differences in the distribution of ability scores have become a research topic of interest since Ellis’s pioneering thesis on *the greater male variability hypothesis*, which posits that men show greater interindividual variability than women do in regard to a wide range of physical and psychological attributes (Ellis, 1894/1934), including intellectual abilities (Hedges and Nowell, 1995; Johnson et al., 2008). By highlighting wider variances for men than women in score distributions, this hypothesis is insightful in terms of understanding why men may outnumber women among the highest and the lowest scoring individuals in samples that show trivial gender differences in mean scores (Hyde, 2014; Reilly et al., 2019).

More recently, research on greater male variability in creativity has also attracted the attention of an increasing number of researchers in the field, who have been puzzled by intriguing findings that showed both gender differences and similarities in creativity (He and Wong, 2011; Kapoor, 2019). Noting the paradoxical patterns of gender differences (especially greater male variability) that tended to emerge in the variability of creativity scores, whereas trivial gender differences (or gender similarities) were often observed in mean comparisons, researchers argued that an understanding of the gender-creativity link could not be complete, due to the lack of variability analyses (see He, 2018). Joining this line of research, the present study aimed to investigate whether men show greater variability than women do in the score distributions of two important constructs of creativity; namely, divergent thinking and creative problem solving.

### The Greater Male Variability Hypothesis

The greater male variability hypothesis (Ellis, 1894/1934), postulating greater male variance in scores or distributions of abilities, provides an important perspective with which to enrich the discourse regarding gender differences, as an alternative to the common understanding of the issue based solely on mean comparisons (Feingold, 1992). In contrast to mean comparisons that concern gender differences in average performance or the central score tendency, the greater male variability hypothesis emphasizes gender differences in (1) the overall distribution and (2) the upper and lower extremes of the distribution, which respectively indicate superior and inferior performance (Hyde, 2014). Because gender differences in variability (despite similar mean scores) imply that the more variable gender will have a higher representation in the higher and/or lower extremes when compared with the less variable gender (Lehre et al., 2008), this line of research may have important educational and political implications, especially for the fields of gifted and special education, in which gender differences in the upper and lower extremes of trait distributions appear to be more critical than those in mean performance (He and Wong, 2014; Reilly et al., 2019).

In terms of operationalization, the greater male variability hypothesis is usually tested with two indexes in the literature. The first index is the *male/female variance ratio (VR)* of the overall score distribution, which is derived by dividing the male variance by the female variance with respect to a given characteristic. A VR greater than 1.0 indicates greater male variability, whereas a VR smaller than 1.0 suggests greater female variability. Furthermore, a VR that equals 1.0 represents equal variabilities in both genders (see Feingold, 1992; Hedges and Nowell, 1995). Using this operationalization^1^, many empirical findings have shown that VRs greater than 1.0 were found in general intelligence (Deary et al., 2003; Johnson et al., 2008), as well as in specific cognitive abilities (He and Wong, 2014). For instance, Feingold (1992) found VRs greater than 1.0 in mechanical reasoning (VR = 1.28), mathematics (VR = 1.20–1.24), and spatial processing (VR = 1.21). Hedges and Nowell (1995) demonstrated VRs = 1.00–1.25 in a wide range of aptitude and achievement tests. He and Wong (2014) also observed that VRs = 1.15–1.62 in gifted characteristics, such as imaginational and intellectual overexcitability (i.e., heightened sensitivity and intensity in imaginational and intellectual ability).

The second index is the gender composition (or the *male/female ratios*) in particular regions of the score distribution for a given psychological characteristic. Greater male variability is represented by an excess of men (e.g., a male/female ratio greater than 1.0) at the high (indicating superior performance) and low (indicating inferior performance) extremes of the score distribution (Deary et al., 2003). For example, researchers reported greater representation of men at both the upper and lower extremes of the IQ score distribution (e.g., Deary et al., 2003; Johnson et al., 2008). Similarly, He and Wong (2014) documented greater male representation at both the upper and lower extremes of the score distribution for intellectual overexcitability (boy/girl ratios = 2.44–2.57) and imaginational overexcitability (boy/girl ratios = 2.07–7.50). Focusing on the upper extreme, Hedges and Nowell (1995) documented that men are more represented in the top 1 to 5% in multiple measures of intellectual ability. Hyde et al. (2008) reported boy/girl ratios of 1.45 and 2.06 in the top 5 and 1%, respectively, of the mathematical score distribution.

### Research Into Greater Male Variability in Creativity

He and Wong (2011) pioneered research into greater male variability in creativity, which is commonly conceptualized as the capability of producing ideas or solutions to problems that are evaluated to be novel and useful (Sternberg and Lubart, 1999). Specifically, they investigated gender differences in creativity by analyzing both means and variability, and found interesting gendered patterns. Based on mean comparisons, they found trivial gender differences in the overall performance of a creative task, as indicated by the total score of the Test for Creative Thinking–Drawing Production (TCT–DP, Urban and Jellen, 1995/2010). However, based on variability analyses, they found empirical support for the greater male variability hypothesis by showing significant gender differences in the overall distribution of the TCT–DP score (VR = 1.62), as well as greater male representation in the upper and lower extremes of the score distribution, in which a boy/girl ratio of 3.40 was found in the upper region. Furthermore, among the low-scoring individuals in the lower region, all of the individuals were boys.

Subsequently, numerous empirical studies have also shown greater male variance in the overall distribution of the creativity scores, as measured by the TCT–DP (e.g., VR = 1.30, He et al., 2013; VR = 1.85–1.88 [except for young children], He et al., 2015; VR = 1.17, Ju et al., 2015; VR = 1.82, Karwowski et al., 2016a; VR = 1.21–1.89, Karwowski et al., 2016b). A review of these studies also suggests that a greater representation of men with a male/female ratio greater than 1.0 might be observed at both or either of the high and low extreme of score distribution. Additionally, greater male variability might occur, regardless of the presence or absence of gender differences in mean scores, implying that the results of variability analyses can be related to or independent of those generated from mean analyses. Hence, researchers have advocated that both variability and mean analyses are necessary in the study of gender differences in creativity, with the aim of generating a more complete picture of the issue from different perspectives (He and Wong, 2011; Karwowski et al., 2016a; He, 2018).

### The Present Study

The present study aimed to examine greater male variability in two important constructs of creativity; namely, divergent thinking and creative problem solving. The rationale is three-fold, with details elaborated upon below.

#### Greater Male Variability in Divergent Thinking and Creative Problem Solving Remains an Under-Investigated Research Question

While a growing body of research has examined greater male variability in creativity, it is interesting to note that all of the empirical investigations (aside from Lau and Cheung, 2015) have been undertaken using one single measure of creativity (i.e., the TCT–DP; Karwowski et al., 2016b; He, 2018). However, in the creativity literature, it is commonly accepted that creativity is a multifaceted construct that can be approached from multiple perspectives and assessed with multiple measures (Rhodes, 1961; Sternberg and Lubart, 1999). Different measures differ in regard to which aspect of creativity they focus on (Agnoli et al., 2016); among the various types of creativity tests, there is variation in the kind of creative process being measured (Haase et al., 2018; Reiter-Palmon et al., 2019).

In Antonietti and Iannello’s (2008) taxonomy, *idea generation*, *combinatory ability*, and *restructuring ability* represent the three key types of creative processes for which distinct creativity tests are usually used as typical measures. For example, idea generation is usually assessed with divergent thinking tests, such as the Torrance Test of Creative Thinking (TTCT; Torrance, 1974) or the Wallach-Kogan Creativity Test (WKCT; Wallach and Kogan, 1965), whereas combinatory ability can be measured with the TCT–DP (Urban and Jellen, 1995/2010). Furthermore, restructuring ability can be tested by creative problem-solving tests such as rebus tests (MacGregor and Cunningham, 2008) or insight problems (e.g., the candle problem; Duncker, 1945).

In this context, it is interesting to note that combinatory ability appeared to have received the most research attention in existing empirical investigations of greater male variability in creativity, in which the TCT–DP has been applied as the predominant measure of creativity (Karwowski et al., 2016b). It is surprising that only one empirical study (Lau and Cheung, 2015) has been documented examining greater male variability in idea generation by applying a divergent thinking test (i.e., the WKCT); this study reported only partial empirical support for the hypothesis. More surprising still, little is known about the generalizability of the hypothesis to the creative process of restructuring ability, owing to the lack of empirical testing on this hypothesis through the assessment of creativity with a creative problem-solving test. To fill this research gap, we aimed to extend the empirical testing of the greater male variability hypothesis in creativity by using a divergent thinking test and a creative problem-solving test.

#### The Divergent Thinking Test and Creative Problem-Solving Test Represent Distinct Types of Creativity Measures Focusing on Different Aspects of Creativity

Divergent thinking refers to the ability to generate diverse and numerous responses to a particular issue that increases the likely output of creative ideas (Guilford, 1956). A typical divergent thinking test involves open-ended problems in different modalities or task content domains (e.g., verbal or figural stimuli), which requires generating as many ideas as possible (Torrance, 1988). Some researchers also suggest using “be-creative” instructions to increase the validity of the measures (Harrington, 1975; Nusbaum et al., 2014). Sample questions include proposing unusual uses for a common object, generating questions about a picture, suggesting ways to improve a product, or completing an incomplete drawing in alternative ways. Divergent thinking is indicated with four main indexes of ability, including (1) fluency (i.e., the ability to generate numerous different responses), (2) flexibility (i.e., the ability to generate different categories of responses, (3) originality (i.e., the ability to generate unusual and unique responses when compared to the norm; see also Wilson et al., 1953, for alternative ways to operationally define originality), and (4) elaboration (i.e., the ability to give elaborative details in the responses; see Kleibeuker et al., 2013).

In Wakefield’s (1989) taxonomy, problem types are differentiated along ill- versus well-defined and open- versus closed-solution dimensions; the divergent thinking test represents a well-defined, open-solution problem. In contrast, the creative problem-solving test represents an ill-defined, closed-solution problem used to assess restructuring ability (Stanciu and Papasteri, 2018). The ability of creative problem solving is usually assessed with insight problems, which stress the role of the “Aha!” experience that leads to the sudden realization of a new approach to a problem as a result of a restructuring of the problem (Wallas, 1926; Weisberg, 2015). In solving this type of problem, participants usually encounter obstacles at first, due to insufficient information or information that was not immediately obvious, which can be used to solve the problem (DeYoung et al., 2008). The key to finding the right solution requires one to reframe his or her mental approach by restructuring the problem when it becomes suddenly clear that the usual approach does not lead to a feasible solution (Beaty et al., 2014). For example, in a typical creative insight problem, such as the candle problem (Duncker, 1945), participants were required to attach a candle to a wall using only the objects that were available. Participants usually went through a process that involved encountering obstacles (e.g., the functional fixedness of a tack box as a container, not a candlestick) at the beginning and then coming up with a sudden “Aha!” solution when a successful reconstruction of the problem representation occurred (e.g., seeing the tack box as a candlestick). The total number of correctly solved insight problems is usually used as an index of creative ability (Weisberg, 2015; Haase et al., 2018).

Individuals’ performances in regard to the measures of divergent thinking and creative problem solving were found to be not correltated (Lin et al., 2012) or weakly correlated (Webb et al., 2017), showing that divergent thinking and creative problem are separable constructs. Moreover, empirical findings also illustrated distinct developmental trajectories of creative problem solving and divergent thinking (Kleibeuker et al., 2013), and creative problem solving was not predicted by divergent thinking, but rather by convergent thinking (Beck et al., 2016).

A summary of the key distinctions between a divergent thinking test and a creative problem-solving test can be found in Table 1.

**TABLE 1:** Key distinctions between a divergent thinking test and a creative problem solving test.

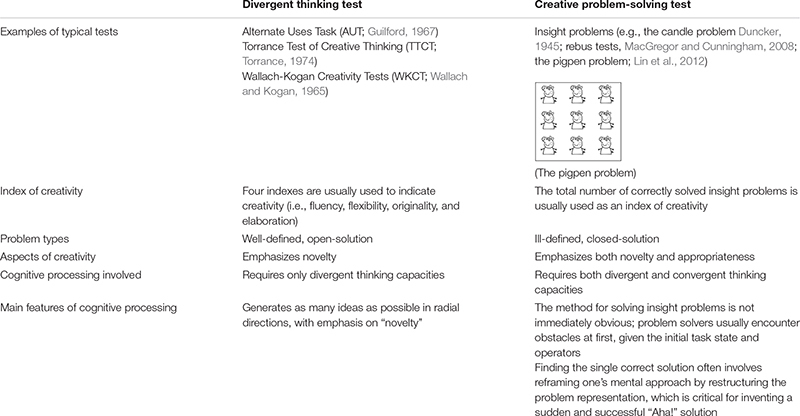

#### Gendered Patterns in Divergent Thinking and Creative Problem Solving Remain Inconclusive Based on the Findings of Mean Comparisons

Mean comparisons remain the sole concern in the existing literature regarding gender differences in divergent thinking and creative problem solving; inconsistent and inconclusive findings have been reported (Abraham, 2016). There are studies showing significant gender differences in favor of women in regard to divergent thinking (e.g., Kuhn and Holling, 2009; Cheung and Lau, 2010) and in favor of men in regard to insightful problem solving (e.g., Jiang et al., 2015). In a direct comparison of the gender-creativity relationship between divergent thinking and creative problem solving, Lin et al. (2012) found similar gendered patterns, which suggest female superiority in divergent thinking and male superiority in creative problem solving.

In spite of some findings that have demonstrated significant gender differences, mixed findings have also been reported that show statistically trivial gender differences, which suggest gender similarities. Perhaps the most striking findings were reported by Baer and colleagues (Baer, 1999; Baer and Kaufman, 2008). In their comprehensive review of studies that compared the mean scores of creative tests between the two genders, they found approximately 50% (Baer, 1999) or even 84% (Baer and Kaufman, 2008) of the reviewed studies reported trivial gender differences, whereas mixed results were found in the remaining studies that showed either female or male superiority (see also Warren et al., 2018). Of note, in most of the documented studies, measurement invariance has been rarely tested prior to the analyses of mean differences, while the establishment of a strict measurement invariance between gender groups was held to be critical to unequivocally allowing mean comparisons (Kuhn and Holling, 2009; Karwowski et al., 2016a).

Given the inconsistent and intriguing gendered patterns reported in divergent thinking and creative problem solving based on the results of mean comparisons, researchers have generally agreed that the empirical findings regarding the gender-creativity link are still far from conclusive; continuing empirical scrutiny is required (Abraham, 2016; He, 2018). Surprisingly, few research findings have been presented on the gendered patterns in the variability of divergent thinking and creative problem solving, as discussed in Section “Greater Male Variability in Divergent Thinking and Creative Problem Solving Remains an Under-Investigated Research Question.” It is thus our intention in the present study to extend this line of research by analyzing gender differences in the distribution of the scores in divergent thinking and creative problem solving, to illuminate the gendered patterns in these aspects of creativity based on an alternative perspective (i.e., the greater male variability hypothesis). Furthermore, issues of measure invariance will be taken into account in the analyses of gender differences in the present study.

#### Hypotheses

Drawing upon the greater male variability hypothesis and relevant research in creativity, it is expected that male participants will demonstrate greater variability than female participants in creativity. Specifically, the following hypotheses were formulated:

H1. Men will show a VR greater than 1.0 in both (a) divergent thinking and (b) creative problem solving;
H2. There will be a male/female ratio greater than 1.0 in the upper and lower extremes of the score distributions in both (a) divergent thinking and (b) creative problem solving.

## Methods

### Participants and Procedure

A convenience sample of 218 (56.0% female) undergraduate students from different academic fields (e.g., arts and languages, education, engineering, sciences, and social studies) were recruited from five universities in Hong Kong to join the study on a voluntary basis. The recruitment of participants from different universities and different academic fields prevented the data from becoming homogenous, thus enabling a better representation of score variance and distribution. An exploration of the thinking process was explained as the main objective of the study. The participants were assured that their participation in the study was completely voluntary, anonymous, and confidential. Written informed consent was obtained from all participants.

The final sample consisted of 206 (51.9% female) participants, with 12 participants excluded from the original sample owing to discontinued participation or incomplete responses to any parts of the measurements (the attrition rate was 5.83%). All participants were ethnic Chinese, with an age range of 18–23 years (*M* = 19.3; *SD* = 1.81). A divergent thinking test and a creative problem-solving test, together with other instruments, were administered to participants in a group setting with standard instructions using the participants’ mother language (i.e., Chinese). Approximately 10–15 participants were tested at a time. The instruments were administered in counterbalanced order. Table 2 presents the demographic statistics of the sample. There were no statistically significant differences between the two gender groups in terms of their age, GPA, education level, or socio-economic background, indicated by their parents’ level of education (all *t*-values ≤ 1.70; all *p*-values > 0.05). The gender composition across the two groups was equivalent (*χ*^2^ = 0.31, *p* = 0.58).

**TABLE 2:** Demographic statistics of the sample.

Measure	Male	Female	*t*	*p*
**Age**				
*M*	19.5	19.1	1.65	0.10
*SD*	1.88	1.73		
**GPA**				
*M*	2.98	3.01	–1.23	0.22
*SD*	0.21	0.11		
**Education (in years)**				
*M*	13.5	13.1	1.70	0.09
*SD*	1.69	1.49		
**Father’s education (in years)**				
*M*	14.1	14.6	–0.89	0.37
*SD*	3.24	3.76		
**Mother’s education (in years)**				
*M*	12.8	13.3	–0.96	0.34
*SD*	3.05	3.15		

**Gender distribution**			***χ*^2^**	***p***

*N*	99	107	0.31	0.58
*%*	48.1	51.9		

### Instruments

#### Divergent Thinking Test

The WKCT (Wallach and Kogan, 1965) was applied to assess divergent thinking in the present study for the following two reasons: (1) It is a commonly used divergent thinking test to assess the creative process of idea generation (Antonietti and Iannello, 2008); and (2) it has well-supported psychometric properties and applicability in Hong Kong Chinese samples (Cheung et al., 2004). The WKCT and its instructions were adapted and translated into Chinese using a back-translation procedure. The initial translation was prepared by a researcher who was bilingual in English and Chinese. The back-translation process was then conducted by two academics who were fluent in both English and Chinese, and were familiar with the literature on creativity. The final translated version was confirmed after discussions and modifications.

The Chinese-adapted WKCT applied in the present study consisted of both verbal and figural test items (Wallach and Kogan, 1965). The verbal test items included instances (“Name as many things as possible that have wheels”) and alternate uses (“Name as many different ways as possible that you could use a newspaper”), and the figural test items included pattern meanings and line meanings, for which respondents were required to name as many things or meanings as possible that the given pattern or line made them think of. Two criteria were applied for the selection of test items. First, they should be items that demonstrated well-supported validity and applicability in Hong Kong Chinese samples based on the study of Cheung et al. (2004). Second, these selected items should have demonstrated a moderate difficulty level (in terms of the number of responses generated) in a pilot study with 50 undergraduate students (54% female; *M*_*age*_ = 19.7 years; *SD* = 2.14 years). Complying with the specifications for administrations of the test (Wallach and Kogan, 1965), the test was conducted in a relaxing, game-like atmosphere, in which participants were given five minutes to respond to each item; hence, a total of 20 min were allowed to complete the WKCT. The participants were assured that there were no model answers and that their responses could be anything they thought of.

The WKCT was scored according to the four indexes of divergent thinking; namely, fluency, flexibility, originality, and elaboration (Kleibeuker et al., 2013). First, fluency was the total number of non-redundant responses provided per participant. Second, flexibility was the number of different categories in which the given responses could be categorized. For instance, in the task “Name as many things as possible that have wheels”, if a participant gave responses such as “a bus, a car, a van, and one’s mind”, he or she would be given a flexibility score of two – one point for responses in the category of transportation and the other point for the response in a non-transportation category for the answer “one’s mind.” Third, originality was rated with reference to all valid responses in the sample, with two points being awarded to responses provided by 1% or less of the respondents and one point to responses provided by 2% to 5% of the respondents (and zero points for responses given by 6% or more of the respondents). The originality ratings of all responses were then summed to reach the ultimate originality score. Finally, elaboration was scored according to the amount of detail provided in a response. Using the same example question about things with wheels, the answer “a taxi” would be given zero points for the elaboration rating, whereas the answer “a red taxi” and “a red taxi speeding down the street” would get an elaboration rating score of one and two, respectively. Similar to the scoring of originality, the ultimate elaboration score was the total of the elaboration ratings of all responses.

All responses were coded by an experienced female researcher in creativity. A second male coder rated 100 protocols to perform an inter-rater reliability assessment. The results of the intraclass correlation coefficients (ICCs) indicated that the test had good inter-rater reliability, with all ICC values being greater than 0.86 (*p* < 0.001; Table 3), suggesting gender has no bias impact on the ratings. Moreover, the measure obtained good internal consistency for the entire sample (*α* = 0.79–0.83, *p*s < 0.01; Table 3), which was comparable to the statistics reported in past studies, supporting the psychometric properties of the test (e.g., *α* = 0.82–0.92 in Cheung and Lau, 2010; *α* = 0.80 in Warren et al., 2018).

**TABLE 3:** Intraclass Correlation Coefficients (ICC) of the WKCT, and the descriptive statistics, Cronbach’s alpha coefficients (α), and correlation coefficients of the two creativity measures.

Creativity measures			*Correlation coefficients (Cronbach’s alpha)*									
	ICC	*M* (*SD*)	1	2	3	4	5	6	7	8	9	10
**Divergent thinking**												
Figural fluency	0.91***	35.5 (10.5)	(0.82**)	0.32**	0.32**	0.33**	0.27**	0.18**	0.18*	0.20*	0.11	0.08
Figural flexibility	0.86***	10.1 (2.99)		(0.80*)	0.31**	0.33**	0.20*	0.24**	0.20*	0.29**	0.18*	0.13
Figural originality	1.00***	7.17 (2.29)			(0.80*)	0.30**	0.18*	0.16*	0.19*	0.22*	0.13	0.11
Figural elaboration	0.89***	39.6 (11.0)				(0.80*)	16*	0.15*	0.18*	0.18*	0.09	0.11
Verbal fluency	0.93***	34.2 (11.1)					(0.81**)	0.32**	0.32**	0.30**	0.08	0.10
Verbal flexibility	0.88***	10.3 (2.94)						(0.83**)	0.31**	0.29**	0.13	0.17*
Verbal originality	1.00***	7.30 (2.62)							(0.80*)	0.30**	0.11	0.13
Verbal elaboration	0.87***	36.6 (10.8)								(0.79*)	0.09	0.12
**Creative problem solving ()**												
Figural CPS	–	60.1 (17.5)									(0.70*)	0.65**
Verbal CPS	–	62.7 (20.2)										(0.71*)

*p < 0.05; **p < 0.01; ***p < 0.001.

#### Creative Problem Solving

The 10 test items of creative problem solving (five verbal problems and five figural problems) used in Lin et al.(2012, pp. 122-123) were employed in the present study for the following three reasons: (1) They showed a moderate difficulty level (with an accuracy rate ranging between 25% and 75%) for samples consisting of Chinese university students; (2) their applicability in Chinese student samples was supported; and (3) they fulfilled the criterion of pure insight problems that require a mental process of reconstruction (Weisberg, 2015). A sample item of the verbal problems, entitled “the magician problem”, was formulated as follows: “A magician claimed to be able to throw a ping pong ball so that it would go a short distance, come to a dead stop, and then reverse itself. He also added that he would not bounce the ball against any object or tie anything to it. How could he perform this feat?” A sample item of the figural problems, entitled “the pigpen problem”, described the task in the following way: “Nine pigs are kept in a square pen. Build two more square enclosures that would put each pig in a pen by itself” (see Table 1 for the figural illustration of the pigpen problem).

The test items were translated into Chinese using a back-translation procedure, as described in Section “Divergent Thinking Test.” Following the procedure of Lin et al. (2012), participants were given 20 min to complete the task in the main study. At the end of the test, participants were also asked to indicate if they had known the answers to any of the problems due to past experience or knowledge. The performance scores for creative problem solving were calculated as the percentage of unfamiliar problems that were answered correctly within the verbal and figural items. The Cronbach’s alpha coefficients obtained in the present sample were 0.71 and 0.70 for the scores for the verbal and figural items, respectively (*p*-values < 0.01; see Table 3).

## Results

### Testing of Assumptions

Several assumptions were examined prior to testing the hypotheses.

#### Correlations Between the Two Measures of Creativity

First, a bivariate correlation analysis was conducted to examine the relationship between the participants’ performance on the divergent thinking test and the creative problem-solving test, in order to determine to what extent these two measures of creativity were correlated with each other. The correlation coefficients shown in Table 3 suggest that individuals’ performance on these two tests was not correlated in most indexes. Among the 18 correlation coefficients, 16 (89%) showed statistically insignificant results (*r*s = 0.08–0.13). In the remaining two coefficients (11%) that showed statistical significance at *p* < 0.05, the extent of the correlation was small; figural flexibility demonstrated a weak correlation with figural creative problem solving (*r* = 0.18), whereas verbal flexibility exhibited a weak correlation with verbal creative problem solving (*r* = 0.17). These results were consistent with those of past studies (Lin et al., 2012; Webb et al., 2017) and implied that the instruments used in this study represented two distinct types of creativity measures.

#### The Confounding Effect of Fluency in Divergent Thinking Scores

Second, because score variances are important in the testing of the greater male variability hypothesis, it is necessary to examine if the fluency scores show overlap of variation (or artifactual correlations) with those of flexibility, originality, and elaboration due to the scoring method applied, given that participants were instructed to generate as many responses as possible in the divergent thinking test (see section “Divergent Thinking Test”). Following Forthmann et al.(2020; p. 99), the below formula was applied to calculate the expected correlations based on the assumption that the average scores of other divergent thinking indexes (e.g., originality divided by fluency) and fluency were independent:

$${\hat{r}}_{T,H}=\frac{\bar{H}}{\bar{T}}\,\frac{s_{T}}{s_{H}}$$

where *H*-bar and *T*-bar denote the sample means and *s* the sample *SD*s, and a $\hat{r}$ greater than 1.0 indicates an overlap (see Table 3 for the sample means and *SD*s). The calculated results revealed that an overlap of variation was observed between fluency and some of the other divergent thinking scores. For the figural domain, a $\hat{r}$ greater than 1.0 was found between fluency and elaboration ($\hat{r}$ = 1.06). For the verbal domain, a $\hat{r}$ greater than 1.0 was found between fluency and flexibility ($\hat{r}$ = 1.14) as well as elaboration ($\hat{r}$ = 1.10). The results appeared to suggest that the correlations between any of these other divergent thinking scores and fluency might be a mix of actual and artifactual correlation (Forthmann et al., 2020). Hence, fluency would be applied as the sole indicator of divergent thinking in the present study for subsequent analyses (Reiter-Palmon et al., 2019).

#### Confirmatory Factor Analysis and Testing of Measurement Invariance

**FIGURE 1:**
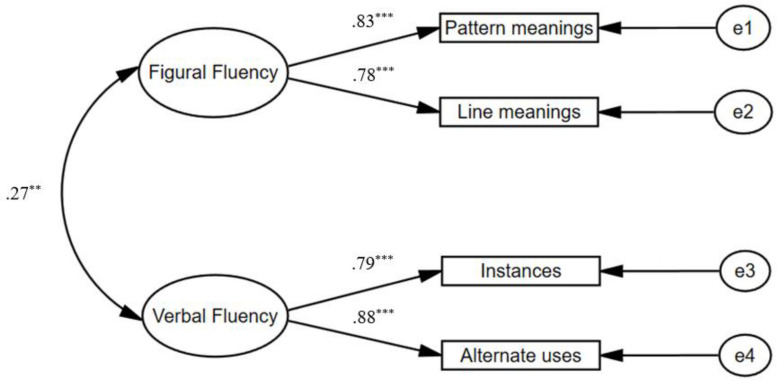
Measurement models for divergent thinking as indicated by figural and verbal fluency. **p < 0.01, ***p < 0.001.

**FIGURE 2:**
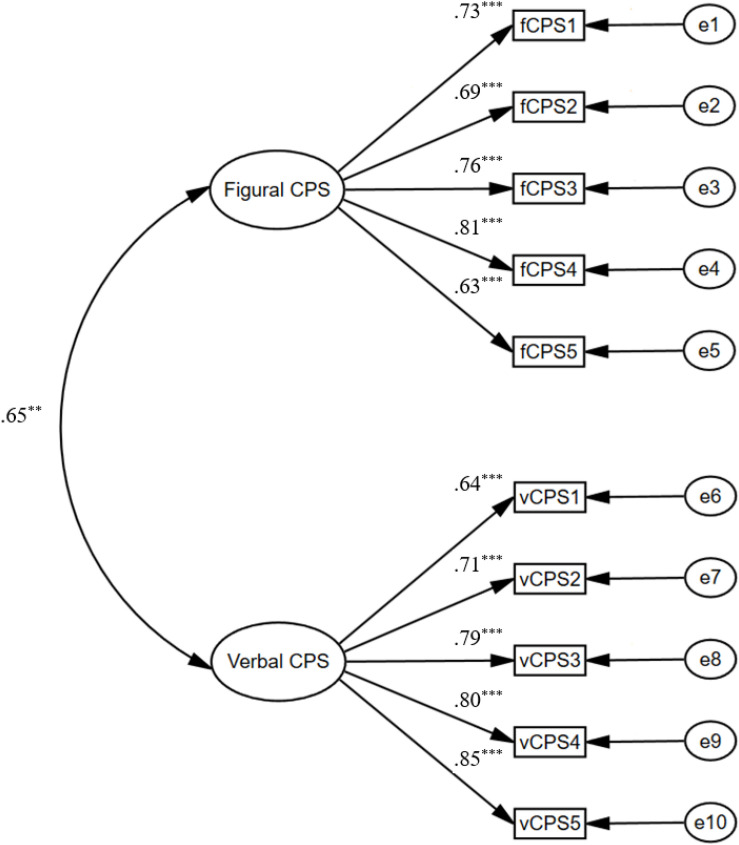
Measurement models for figural and verbal creative problem solving (CPS). **p < 0.01, ***p < 0.001.

Third, a confirmatory factor analysis (CFA) was performed to investigate the construct validity of the divergent thinking and the creative problem solving. For divergent thinking, a two-factor model was tested, with two items loading on figural or verbal fluency (see Figure 1). The fit indexes of the resulting model (*CFI* = 0.9471, *TLI* = 0.9562, *RMSEA* = 0.0463, *SRMR* = 0.0455, *χ*^2^ = 12.9, *df* = 1, *p* < 0.01) were regarded as acceptable. With respect to creative problem solving, a two-factor model was tested, with five items loading on figural or verbal creative problem solving (see Figure 2). The results of the fit indexes also suggest that the model fit the data well (*CFI* = 0.932, *TLI* = 0.920, *RMSEA* = 0.051, *SRMR* = 0.049, *χ*^2^ = 85.1, *df* = 34, *p* < 0.01).

To further test whether the construct validity of the scale is equally supported in both genders, we followed the procedures suggested by Vandenberg and Lance (2000) to test measurement invariance, with progressively restrictive stages. First, a configural invariance test was performed to establish a baseline model across the two gender groups, in which factor loadings, intercepts, and residuals were allowed to be estimated freely. Second, the metric invariance model was tested, in which all factor loadings were constrained the same across the groups. Finally, the scalar invariance model was tested, in which the factor loading and indicator intercepts were contrained to be the same across groups. Chen’s (2007) recommendations of cutoff points for testing invariance were applied: for testing loading invariance, Δ*CFI* ≤ −0.005 supplemented by Δ*RMSEA* ≥ 0.010 or Δ*SRMR* ≥ 0.025 would indicate non-invariance; for testing intercept or residual invariance, Δ*CFI* ≤ −0.005 supplemented by Δ*RMSEA* ≥ 0.010 or Δ*SRMR* ≥ 0.005 would indicate non-invariance (p. 501).

The results of the fit statistics tended to support the idea that the structure of the scale was invariant across the two genders for both divergent thinking (configural invariance: *CFI* = 0.9462, *TLI* = 0.9560, *RMSEA* = 0.0471, *SRMR* = 0.0468; metric invariance: *CFI* = 0.9452, *TLI* = 0.9557, *RMSEA* = 0.0479, *SRMR* = 0.0648, scalar invariance invariance: *CFI* = 0.9444, *TLI* = 0.9556, *RMSEA* = 0.0559, *SRMR* = 0.0688) and creative problem solving (configural invariance: *CFI* = 0.9314, *TLI* = 0.9201, *RMSEA* = 0.0499, *SRMR* = 0.0505; metric invariance: *CFI* = 0.9304, *TLI* = 0.9204, *RMSEA* = 0.0544, *SRMR* = 0.0545; scalar invariance invariance: *CFI* = 0.9294, *TLI* = 0.9194, *RMSEA* = 0.0553, *SRMR* = 0.0546). The results regarding the strong invariance supported the idea that the factor means and variances could be applied in the subsequent analyses of gender differences.

### Variability Analyses

To test H1 and H2, variability analyses were performed on the factor scores of divergent thinking (using fluency as the indicator) and creative problem solving by applying the two operational indexes of greater male variability, including the use of male/female VR to test H1, and the use of male/female ratios in the upper and lower tails of score distributions to test H2. In relation to VR, *F*-tests of equality of variance were used to test for significant differences in the heterogeneity of the variances between the two genders. With respect to the male/female ratios in the distribution tails, chi-square tests were employed to test for significant differences in gender proportions in particular regions of the distribution. The results of the variability analyses are summarized in Table 4.

**TABLE 4:** Results of gender differences in factorial means and variability of divergent thinking (DT) and creative problem solving (CPS).

	Factor means (variances)				Male/female ratio in tails (*χ*^2^)			
	Male (*n* = 99)	Female (*n* = 107)	*d* (*t*-value^*a*^)	*VR* (*F*-value)	Lowest 5%	Lowest 10%	Top 10%	Top 5%
Figural DT	34.6 (148.6)	36.6 (86.7)	−0.18 (−1.75)	1.71 (5.68*)	4.26 (4.09*)	1.69 (1.33)	2.54 (1.93)	6.82 (6.15**)
Verbal DT	30.9 (106.3)	33.3 (107.5)	−0.23 (−2.72)	0.99 (2.67)	5.84 (6.22*)	4.25 (4.53)	0.98 (0.01)	0.78 (0.26)
Figural CPS	60.5 (358.1)	56.0 (203.5)	0.27 (3.37)	1.76 (5.92*)	4.79 (5.02*)	2.46 (2.34)	2.54 (1.25)	5.32 (5.98*)
Verbal CPS	62.4 (494.3)	61.0 (322.1)	0.07 (0.26)	1.53 (5.16*)	5.32 (5.98*)	3.25 (3.49)	0.95 (0.02)	1.08 (0.01)

*p < 0.05, **p < 0.01. ^*a*^Bonferroni procedures were used to adjust for multiple comparisons.

#### Results of VRs

As shown in Table 4, the predicted pattern of VRs greater than 1.0 was observed in figural divergent thinking (VR = 1.71) but not in verbal divergent thinking (VR = 0.99). The results of *F* tests of equality of variance illustrated that men showed significantly larger variances than women in figural divergent thinking (*F* [1, 204] = 5.68, *p* < 0.05). These results suggest that H1 was supported in the figural domain, but not in the verbal domain, of divergent thinking. For creative problem solving, a VR greater than 1.0 was observed in both of the figural (VR = 1.76) and verbal (VR = 1.53) domains, lending support to H1. The results of *F* tests of equality of variance indicated that significantly larger variances were found for men than for women in both figural and verbal creative problem solving (*F* [1, 204] = 5.16–5.92, *p* < 0.05).

#### Results of Male/Female Ratios in the Distribution Tails

With respect to the results of the male/female ratio in the extremes of the score distributions, as presented in Table 4, the expected pattern of H2 was observed in the lowest 5% to 10% of the regions of score distribution for both divergent thinking (male/female ratio = 1.69–5.84) and creative problem solving (male/female ratio = 2.46–5.32) in the figural and verbal domains. The results of chi-square tests further revealed that significantly more men than women were represented in the lowest 5% of the score distribution of divergent thinking and creative problem solving (*χ*^2^ values ≥ 4.09, *p*-values < 0.05).

Focusing on the top 5% to 10% of the regions, inconsistent results were found. On the one hand, H2 was supported in the figural domain of divergent thinking and creative problem solving, in which a male/female ratio greater than 1.0 was found for the top 5% to 10% of the score distribution (divergent thinking: male/female ratio = 2.54–6.82; creative problem solving: male/female ratio = 2.54–5.32). On the other hand, mixed results were observed for the verbal domain, in which the predicted pattern of H2 was only observed for the top 5% of the score distribution of verbal creative thinking (male/female ratio = 1.08). A contradictory pattern (i.e., a male/female ratio < 1.0) was found for the top 5% to 10% of the score distribution for verbal divergent thinking (male/female ratio = 0.78–0.98), and the top 10% of the score distribution of verbal creative problem solving (male/female ratio = 0.95), which suggests greater female representation in these regions. The results of the chi-square tests revealed that significantly greater male representation was found in the top 5% of the regions for figural divergent thinking and figural creative problem solving (*χ*^2^ values ≥ 5.32, *p*-values < 0.05).

### Mean Analyses

Because gender differences in variability could be related to or independent of mean differences (Arden and Plomin, 2006), and both variability and mean analyses are necessary to generate a more complete picture with respect to the understanding of the gender-creativity link (He and Wong, 2011; Karwowski et al., 2016a; He, 2018), we also performed a multivariate analysis of variance (MANOVA) to examine whether there were gender differences in the factor means of divergent thinking and creative problem solving. The results of *t*-values and Cohen’s *d*s, summarized in Table 4, suggest a general pattern of trivial gender differences in the mean scores, in which no statistically significant gender differences were found for all factor means of divergent thinking and creative problem solving (*t*-values ≤ 3.37; *n.s*.). Cohen’s *d*s ranged between 0.07 and 0.27, suggesting that the effect sizes were near zero or were small (Cohen, 1988).

## Discussion

Adding to previous research regarding greater male variability in creativity, in which the TCT–DP has been applied as the predominant measure of creativity (He and Wong, 2011; Karwowski et al., 2016b; He, 2018), the present study extended this line of research to examine greater male variability in creativity by using two further measures of creativity: a divergent thinking test and a creative problem-solving test. Extending the study of greater male variability by using distinct measures of creativity may enrich this line of research by: (1) displaying the gendered patterns of distributions of various creativity scores that feature distinct aspects of creativity; and (2) validating the greater male variability hypothesis in creativity in a more comprehensive way through measuring the construct based on multiple psychometric approaches.

Three interesting findings were observed. First, our results of VRs revealed a general pattern that men show greater variances than women in creativity. Second, by using creativity instruments that consist of both figural and verbal test items, our results further illustrated domain-specific gendered patterns in the distribution of creativity scores, which suggest that greater male variability was more likely to occur, and with a larger effect size, in the figural domain than in the verbal domain of divergent thinking and creative problem solving. Third, our results regarding gender composition (i.e., male/female ratios) in the extremes of the score distributions also suggest interesting domain-specific gendered patterns in the upper tails, despite a consistent pattern of greater male representation in the lower tails of the distribution. More details of these findings are discussed below.

### The Results of VRs Tended to Support Greater Male Variability

Our results of VRs tended to support a general pattern of greater male variance in the overall distribution of the creativity scores in both divergent thinking and creative problem solving, with 75% of the obtained VRs in divergent thinking and creative problem solving showing a value greater than 1.0. Previous studies that investigated greater male variability in creativity using the TCT–DP reported similar findings that 73% to 100% of the obtained VRs were above 1.0 (e.g., He and Wong, 2011; He et al., 2013, 2015; Ju et al., 2015; Karwowski et al.,2016a,b).

The empirical findings regarding the relatively consistent pattern of VRs above 1.0 were also in line with the findings generated from large-scale meta-analyses that examined the greater male variability hypothesis based on national or international data drawn from representative samples. For example, Feingold (1992) reported an overall rate of 86% of VRs greater than 1.0 in four norming studies of aptitude tests. In large national samples, Nowell and Hedges (1998) found that 95 to 100% of the VRs were greater than 1.0 in the mental test scores. Other researchers also found that 93 to 95% of the VRs greater than 1.0 in the analyses of more recently available data collected with international assessments, including the Programme for International Student Assessment (PISA), the Progress in International Reading Literacy Study (PIRLS), and the Trends in International Mathematics and Science Study (TIMSS; Bayer and Monseur, 2016; Gray et al., 2019). Joining this body of research, our results of VRs in divergent thinking and creative problem solving lent further empirical support to the greater male variability hypothesis postulated by Ellis (1894/1934).

The consistent observation regarding greater male variance in the overall distribution in ability scores is interesting, despite increasing evidence showing greater gender similarities (Hyde et al., 2008; Hyde, 2014) or even female superiority in some cases (Lietz, 2006; Gray et al., 2019). In the present study, the results of mean comparisons of the scores of divergent thinking and creative problem solving also showed a pattern of gender similarity, with Cohen’s *d*s ranging between 0.07 and 0.27, suggesting that the effect sizes were near zero or were small (Cohen, 1988). These findings buttress the methodological stance that highlights the necessity of including both mean and variability analyses to generate a more complete picture regarding gender differences (He and Wong, 2011; He, 2018).

### Domain-Specific Gendered Patterns Found in Overall Score Distributions

The findings of this study also revealed a domain-specific gendered pattern in VRs, which enriches the discourse regarding the greater male variability hypothesis in creativity by showing the gender effect regarding greater male variability in terms of (1) the probability of occurrence and (2) the magnitude or the effect size of the difference. Referring to the probability of occurrence, our VR results suggest that greater male variability appeared to be more likely to occur in the figural modality of creativity, in which 100% of the VRs for the figural domain of divergent thinking and creative problem solving had a value greater than 1.0. However, for the verbal modality of such creativity, only 50% of the VRs exhibited a value greater than 1.0.

With reference to effect size, we followed Feingold’s (1992) rule of thumb with respect to the interpretation of the magnitude regarding the gender difference in variance. For example, a VR of 1.20 would indicate that men showed larger variance than women for 20% of the effect size, and an average of several VRs (e.g., 1.17 = [1.40 + 1.10 + 1.01]/3) indicates that men generally showed larger variance than women by 17%. By applying Feingold’s (1992) method, we found that men, on average, showed 74% more variance than women in the figural domain of divergent thinking and creative problem solving. However, relatively smaller effect sizes were found for the verbal domain in regard to such creativities, in which men, on average, showed 26% more variance than women. The domain-specific findings of the present study with respect to the occurrence and the effect size of greater male variability are in congruence with the results reported in Lau and Cheung (2015), which examined greater male variance in creativity by using a divergent thinking test (i.e., the WKCT). Lau and Cheung (2015) also found that greater male variability was more likely to be observed in responses to figural stimuli than to verbal stimuli, while equal variability or greater female variability were more likely to be observed in responses to verbal stimuli.

Interpreting the domain-specific findings of the present study was interesting in light of the results of previous studies that have examined greater male variability using the TCT–DP. Owing to its nature of assessing creativity via performance in drawing production, which is completed by any combination of the six given figural fragments (Urban and Jellen, 1995/2010), the TCT–DP is regarded as a figural form of creativity test (Urban, 1991, 2004; Mullineaux and Dilalla, 2009). Past studies applying the TCT–DP generally documented a relatively high occurrence (i.e., 73% to 100%) of the obtained VRs being greater than 1.0 and a relatively large effect size, showing that men displayed more variance than women did, with a range between 17% and 89% (VR = 1.30, He et al., 2013; VR = 1.85–1.88 [except for young children], He et al., 2015; VR = 1.17, Ju et al., 2015; VR = 1.82, Karwowski et al., 2016a; VR = 1.21–1.89, Karwowski et al., 2016b). Greater male variability found in the figural domain of creativity of the present study was in line with the above empirical findings using the TCT-DP. The measures applied in the present study enabled us to take a further step in directly comparing the patterns of creativity scores in figural and verbal forms. The current findings regarding the VRs in the scores of divergent thinking and creative problem solving, when considered simultaneously, appeared to suggest the conclusion that modalities (verbal and figural) of creativity have an impact on the gendered patterns of variability, in which greater male variability was more likely to be observed in the figural domain of creativity, with larger effect sizes, when compared with the verbal domain.

Intriguingly, the domain-specific gendered pattern in variance appeared not to be an unusual phenomenon in the literature regarding gender differences in cognitive ability, in regard to which men usually showed greater variability in quantitative, spatial, and mathematical abilities, but showed equal variability in verbal ability, compared to women (Maccoby and Jacklin, 1974; Feingold, 1992; Strand et al., 2006; Lohman and Lakin, 2009). Our research concurs with previous studies that have shown greater male variability using the WKCT (Lau and Cheung, 2015) and the TCT–DP (He and Wong, 2011; He et al., 2013, 2015; Ju et al., 2015; Karwowski et al.,2016a,b); together, our research and previous studies provide empirical support to expand the list of cognitive abilities by including creativity, which may show domain-specific patterns in the differences in gender variability. Altogether, these findings seem to suggest that greater male variability may not be a uniform and unitary phenomenon across all sorts of human cognitive abilities. A simple solution may not be sufficient to address the complex issue regarding gender differences in variability. Whether or not, or how well, the greater male variability hypothesis is supported would depend on the modalities of the test content used and the responses required, as well as the domain of the abilities of concern.

### Domain-Specific Gendered Patterns in the Upper Tails of the Distributions

Another important finding of the present study concerns the domain-specific gendered patterns regarding gender composition (i.e., male/female ratios) in the tails of the creativity score distributions. Overall, the findings appeared to suggest that the greater male variability hypothesis was relatively well supported for the figural domains of divergent thinking and creative problem solving, in which more men than women were represented in both the upper and lower extremes of score distributions, corroborating the prediction of the hypothesis. However, for the verbal domain, greater female representation and equal representation of the two genders may occur in some upper regions, despite the predicted pattern of greater male representation being consistently supported in the lower regions of these verbal forms of creativity. These findings may be helpful in explaining why greater male variability was more likely to be observed in the figural domain of creativity with larger effect sizes in VRs when compared with the verbal domain. For the figural domain, greater male representation was consistently observed in both the lower and upper regions. However, for the verbal domain, although greater male representation was consistently observed in the lower regions, it was not consistently observed in the upper regions, where other gendered patterns, such as greater female representation or equal representation of the two genders might also appear, resulting in compensation for the overrepresentation of men in the lower regions and consequently reducing the effect size of greater male variability for verbal forms of creativity.

The inconsistent gendered patterns found in the upper regions, compared to the consistent greater male representation in the lower regions of verbal creativity scores, were interesting. Do these results imply a possibility of a male disadvantage, a female advantage, or gender equality in verbal functioning? Relevant to this speculation, Strand et al. (2006) observed that greater representation of men was found only in the bottom 5% (but not the top 5%) of the score distributions for verbal reasoning. In contrast, for quantitative and non-verbal reasoning scores, greater representation of men was found at both the top and bottom 5%. Strand et al.’s (2006) findings imply that an overrepresentation of men could be observed among both high-scoring and low-scoring individuals for quantitative and non-verbal reasoning abilities. However, for verbal reasoning abilities, excess of men could only be observed among low-scoring individuals, which appears to imply a male disadvantage in verbal functioning.

Similar results suggesting a male disadvantage in verbal functioning were also reported in Hedges and Nowell (1995), which documented an overrepresentation of men in the bottom 10% of participants’ reading comprehension ability. There are also research findings suggesting greater male representation in populations with reading impairment (e.g., dyslexia; Hawke et al., 2009). Interestingly, a different picture was observed for advanced readers who demonstrated relatively superior performance, in which far more women than men attained the highest level of language proficiency (Reilly et al., 2019). The findings regarding the domain-specific gendered patterns in the upper tails of the creativity score also seemed to corroborate the observation regarding high levels of creative accomplishment; although more men than women pursued domains of invention in science, musical composition, and painting, the prevalence of men and women in expressive domains, such as writing and drama, was comparable (Runco, 1986; Kaufman, 2006).

In spite of the findings regarding the domain-specific gendered patterns in the score distributions of figural and verbal creativity, limited information is available about why such patterns occur. Recent neural scientific studies suggest a possible mechanism by which a female advantage in verbal creativity might be related to the greater inter-hemispheric connections or integrations in women’s brains (Fink and Neubauer, 2006; Abraham, 2016). Whereas high levels of visuospatial (or figural) divergent thinking were related to stronger engagement of the right hemisphere, high levels of verbal divergent thinking performance were more related to the interaction or integration between the left and right hemispheres (Faust and Kenett, 2014; Kenett et al., 2015; Chen et al., 2019). Greater inter-hemispheric connections or integrations in women’s brains may result in greater female representation at the higher end of the verbal creativity score distribution (Chen et al., 2019).

Other researchers have also highlighted the possible contribution of socio-cultural factors (Feingold, 1994; Cheung and Lau, 2010) or the interplay of biological/evolutionary and socio-cultural factors (Vernon, 1989; Abra and Valentine-French, 1991; Wood and Eagly, 2002). In line with the socio-cultural perspective, Gray et al. (2019) noted that greater male variability was not universally homogenous and that quantifiable differences exist among nations. They also argued that some heterogeneity could be attributed to social practices or policies that target increasing male-female quality and general male-female performance (p. 27). Relevant to this argument, there are research findings showing that teachers are one of the main sources that shape students’ creative self-efficacy, which in turn influences creative outcomes (Karwowski, 2011; Du et al., 2020); the teacher effect was stronger among female than male students (Karwowski et al., 2015). Moreover, domain-specific analyses showed that men tended to have higher levels of self-efficacy than women in math and science-analytic creativity (Kaufman, 2006; Karwowski et al., 2015) and problem solving (Hughes et al., 2013), whereas women showed higher levels of creative self-efficacy than men in the arts and in language (Kaufman, 2006; Hughes et al., 2013; Karwowski et al., 2015).

Put differently, research findings appear to imply that both biological and socio-cultural factors may contribute to the observed domain-specific gendered patterns in the variability of figural and verbal creativity. Multiple theoretical perspectives should be taken into account, with an aim of understanding the complex mechanisms that contribute to the intriguing gendered patterns of creativity (Hyde, 2014; He, 2018).

### Limitations and Future Research

Some limitations of this study should be noted. The first limitation concerns the measures of creativity. Whereas, at the conceptual and theoretical levels, both divergent thinking tasks and creative insight problems are regarded as important indexes of creative potential (Stanciu and Papasteri, 2018), the predictive power of the WKCT and the creative problem-solving test for real-life creative behaviors and achievement is still an issue undergoing debate (Zeng et al., 2011; Beaty et al., 2014). Hence, the findings obtained in this study may have limitations with regard to gender differences in real-life creativity. In future research, the Torrance Tests of Creative Thinking (TTCT) could be used as an alternative option in the research of greater male variability, given that compelling evidence has supported its predictive power for real-life creative achievement based on longitudinal follow-up studies of 50 years (Runco et al., 2010).

Second, the variability analyses of divergent thinking in the present study was based on the fluency score as the sole indicator, given that a confound effect of fluency was found in flexibility, originality, and elaboration. Adopting fluency as the sole criterion in the analysis is limited in the sense that it only illustrated the quantity of divergent production but failed to capture the quality of divergent thinking (see also Forthmann et al., 2020). As suggested by Reiter-Palmon et al. (2019), it is desirable to consider alternative scoring methods in the application of divergent thinking tests.

Third, the creativity measures employed to study greater male variability are still limited in assessing specific aspects of creativity in relation to creative thinking, such as idea generation, combinatory ability, and restructuring ability (Antonietti and Iannello, 2008). Under the multiple-measurement approach to the multifaceted concept of creativity (Rhodes, 1961; Sternberg et al., 2005), future research should consider other measures of creativity that focus on alternative aspects of creativity, such as creative personality and creative self-efficacy (Haase et al., 2018).

Fourth, the sample in the present study was limited to Chinese university students. Past research suggests that greater male variability was supported in adolescents and emerging adults, but not in young children, among which greater female variability was found (He et al., 2015; He, 2018). In this regard, future studies should explore whether the research findings of the present study can be generalized to other age groups. Future studies should also explore whether the research findings can be generalized to other samples with different cultural, educational, or ethnic backgrounds.

## Conclusion

Despite the abovementioned limitations, the findings derived from the present study enrich the current understanding of gender differences in creativity, as well as the discourse surrounding greater male variability. Bearing in mind the domain-specific patterns in the occurrence and effect size of gender differences in variability, the consistent observation regarding greater male variance in the overall distribution in creativity and other ability scores is interesting and worthy of our attention, especially in the context of contemporary society, which is committed to promote gender equity, particularly within the sphere of educational opportunity (see also Gray et al., 2019). The research findings of the present study further imply that special consideration should be given to the differences in creativity performance pertaining to gender in the tails of the distributions and in different domains, which may have important implications on educational policies and practices. These findings also illustrate the desirability for continuing empirical scrutiny with respect to greater male variability in creativity as a multifaceted construct.

## Data Availability Statement

Requests to access the datasets should be directed to the corresponding author.

## Ethics Statement

The studies involving human participants were reviewed and approved by The Human Research Ethics Committee of the Education University of Hong Kong. The participants provided their written informed consent to participate in this study.

## Author Contributions

W-JH contributed to the conception and design of the work as well as the acquisition, analysis, and interpretation of the data. Both authors contributed to the article and approved the submitted version.

## Conflict of Interest

The authors declare that the research was conducted in the absence of any commercial or financial relationships that could be construed as a potential conflict of interest.

## Footnotes

1 This operationalization depends on which variance is used as numerator or denominator. The operationalization used in this study is to facilitate the interpretation of a result in relation to the hypothesis (i.e., greater male variability).
